# The resilience–distress paradox in HIV: latent profiles reveal that resilience compensates for low mindfulness

**DOI:** 10.3389/fpubh.2026.1950568

**Published:** 2026-10-12

**Authors:** Fang Chen, Xixi Yan, Xiaomin Ding, Juan Li, Shihua Pan, Ling Feng, Xiaoxia Zhang, Yueling Wu, Huimin Sun, Hongyun Xu

**Affiliations:** 1 Department of Neurology, Zhongnan Hospital of Wuhan University, Wuhan, China; 2 Department of Allergy, Zhongnan Hospital of Wuhan University, Wuhan, China; 3 Department of Geriatrics, Zhongnan Hospital of Wuhan University, Wuhan, China; 4 Medical Social Work Department, Zhongnan Hospital of Wuhan University, Wuhan, China; 5 Department of General Medicine, Zhongnan Hospital of Wuhan University, Wuhan, China; 6 Department of Infectious Diseases, Zhongnan Hospital of Wuhan University, Wuhan, China; 7 Department of Scientific Research, Zhongnan Hospital of Wuhan University, Wuhan, China; 8 Department of Neuropsychology, Zhongnan Hospital of Wuhan University, Wuhan, China; 9 Party Committee Office, Zhongnan Hospital of Wuhan University, Wuhan, China

**Keywords:** compensatory interaction, HIV/AIDS, latent profile analysis, mindfulness, psychological distress, quality of life, resilience

## Abstract

**Background:**

People living with HIV (PLWH) face elevated risks for anxiety and depression, yet not all develop clinically significant distress—a phenomenon we term the resilience-distress paradox, whereby individuals with high psychological resilience (the capacity to adapt positively to adversity) nonetheless experience severe distress. Drawing on a person-centered approach, this study identified distinct psychological adaptation profiles based on mindfulness, resilience, anxiety, and depression, and examined their associations with quality of life, social support, and sociodemographics.

**Methods:**

A cross-sectional survey of 403 people living with HIV (mean age 35.5 years; 86.1% male) was conducted. Latent profile analysis (LPA) was based on the Mindful Attention Awareness Scale, the Brief Resilience Scale, the GAD-7, and the PHQ-9. Analysis of variance and chi-square tests examined profile differences in quality of life, social support, and clinical variables; hierarchical regressions tested the mindfulness × resilience interaction on distress.

**Results:**

Three profiles emerged: Well-Adapted (52.4%), Moderate (39.7%), and Distressed (7.9%). Mindfulness showed a strict gradient across profiles (all *p* <0.001), whereas resilience did not differ between the Moderate and Distressed profiles (*p* = 0.073, *d* = 0.44). Quality of life followed the same gradient. Perceived social support was higher in the Well-Adapted than in the Moderate profile, but neither profile differed significantly from the Distressed profile. Sexual orientation distribution also differed across profiles: heterosexual individuals were significantly underrepresented and homosexual individuals significantly overrepresented in the Distressed profile, whereas identity-uncertain individuals showed only a non-significant trend toward overrepresentation.

**Conclusion:**

PLWH exhibit substantial heterogeneity in psychological adaptation. Interaction analyses revealed that resilience buffered anxiety and depression at all levels of mindfulness, with the strongest protection where mindfulness was lowest. This compensatory pattern, rather than a threshold effect, suggests that resilience can partially substitute for limited mindfulness, whereas the absence of both resources creates the greatest vulnerability. These findings support stratified interventions tailored to individuals' resource profiles.

## 1 Introduction

The widespread availability of combination antiretroviral therapy has transformed HIV infection into a manageable chronic condition, with life expectancy among people living with HIV (PLWH) now approaching that of the general population (1). Paradoxically, despite these therapeutic advances, the psychological burden of HIV has not declined in parallel: depressive symptoms among PLWH are approximately seven times as prevalent as in the general population (31% vs. 4.4%) (2, 3), and clinical anxiety nearly eight times (29% vs. 3.6%) (3, 4). More striking is the divergence among PLWH exposed to similar stressors—a considerable proportion exhibit posttraumatic growth (5), whereas others develop persistent, clinically significant distress (6). We term this asymmetric response the resilience–distress paradox.

Traditional linear stress–diathesis models fail to account for this paradox: if psychological risk were simply a linear function of stressors or a sum of protective factors, outcomes would vary continuously across the population. Instead, we observe discrete qualitative divides, suggesting nonlinear dynamics that remain poorly understood (7, 8).

In the literature on HIV psychological adaptation, two core protective factors have emerged: mindfulness and resilience. Mindfulness—defined as the intentional, nonjudgmental awareness of present-moment experience—has been consistently linked to lower depression, reduced perceived stress, and greater positive affect among PLWH (9). Resilience, in turn, refers to the capacity to adapt, recover, or even grow in the face of adversity, encompassing emotion regulation, problem-solving, and interpersonal support (10). Although these constructs differ—resilience as an ability or outcome versus mindfulness as a skill or process—they are known to covary and interact (11, 12); however, the precise nature of this interaction remains unresolved. One possibility is a synergistic, threshold-like pattern: mindfulness may provide the attentional stability and decentering capacity needed to effectively deploy resilience strategies (13, 14), such that resilience confers stronger protection when mindfulness is high. Alternatively, a compensatory pattern may operate: mindfulness and resilience may act as partially substitutable resources (15, 16), with resilience compensating when mindfulness is low—and thus offering stronger protection at low levels of mindfulness. These competing hypotheses have yet to be tested directly in PLWH.

Most studies of psychological adaptation in PLWH have adopted a variable-centered approach, using regression or structural equation modeling to examine how protective factors relate to mental health outcomes. These studies have shown that mindfulness and resilience, both independently and jointly, predict lower distress in PLWH (14, 17). However, they share a fundamental limitation: they implicitly assume population homogeneity, treating all individuals as if they followed the same statistical regularities and are reducible to an “average person” (18). Fisher et al. showed that within-person variability was two to four times larger than between-person variability—thus, group-level averages capture only a fraction of individual-level variability, and conclusions drawn from group-level data can be misleading when applied to individuals (18). For the resilience–distress paradox—where some PLWH maintain wellbeing while others deteriorate under the same disease-related stressors—this limitation is not merely theoretical: it directly undermines our ability to explain why individuals with similar levels of protective factors can show markedly different outcomes.

Heterogeneity is therefore a more plausible feature of human functioning than the uniformity implied by group averages—a premise central to person-centered research (8). Among PLWH, latent profile analysis (LPA) has revealed multiple distinct psychological configurations (19–22). Of particular note, Boga and Dale identified four adversity–resilience profiles in Black American women with HIV: between two profiles with comparably high resilience, the high-adversity profile showed significantly worse outcomes on several mental health indicators—most notably, more severe posttraumatic stress symptoms—than the low-adversity profile, although depressive symptoms did not differ significantly between them (23). This divergence demonstrates that equivalent levels of resilience can yield markedly different psychological outcomes depending on the configuration of co-occurring protective and risk factors—a pattern incompatible with a purely linear, additive interplay between mindfulness and resilience. These findings thus indicate that the relationship between protective factors is nonlinear; a person-centered approach is therefore needed to identify the latent configurations underlying the resilience–distress paradox.

To adjudicate between these possibilities, the present study employed LPA to identify distinct psychological adaptation profiles among PLWH, using mindfulness, resilience, anxiety, and depression as indicators—a combination not previously examined within a single LPA model. We then validated the resulting profiles against external criteria: quality of life (WHOQOL-HIV BREF), perceived social support (MSPSS), and key sociodemographic and clinical variables, consistent with prior profile-analytic studies of psychological adaptation in PLWH (22, 23).

Building on this framework, we derived specific expectations for the profile structure and its external validation. Because the resilience–distress paradox reflects a polarization between robust adaptation and severe distress, we expected profiles to be ordered along a severity gradient, from a Well-Adapted group (high mindfulness and resilience, low distress) through a Moderate group to a Distressed group (low protective resources, clinically elevated distress), consistent with prior latent profile studies of PLWH (19, 20). For external validation, we anticipated a corresponding monotonic gradient in which the Well-Adapted profile reports the highest quality of life and social support, the Moderate profile intermediate levels, and the Distressed profile the lowest, as quality of life and social support are established downstream indicators of psychological adaptation in PLWH (24, 25). Regarding sexual orientation, minority stress theory holds that sexual-minority individuals face chronic, stigma-related stressors that elevate risk for psychological distress (26, 27); we therefore predicted that sexual-minority participants would be overrepresented in the Distressed profile. If the compensatory pattern holds, we would additionally expect resilience to buffer distress most strongly at low mindfulness levels.

Specifically, we tested three hypotheses. First, the profile-existence hypothesis predicted that at least three distinct profiles would emerge, consistent with prior latent profile studies of PLWH that have identified multiple adaptation or distress profiles along a severity gradient (19, 20). Second, the mindfulness–resilience interaction hypothesis predicted a significant mindfulness × resilience interaction on anxiety and depression, with the direction of this interaction—whether synergistic (stronger protection from resilience at high mindfulness) or compensatory (stronger protection at low mindfulness)—to be determined empirically. Third, the external-validity hypothesis predicted that profile membership would be associated with gradients in quality of life and social support, and that sexual-minority status would be associated with membership in the Distressed profile.

## 2 Methods

### 2.1 Participants and procedure

This cross-sectional study employed convenience sampling to recruit PLWH receiving treatment at Zhongnan Hospital of Wuhan University and Wuhan Jinyintan Hospital in Wuhan, Hubei Province, between December 2025 and March 2026. Participants were recruited from both inpatient wards and outpatient clinics. Trained research assistants approached eligible patients during clinical visits, explained the study purpose, and obtained informed consent. Participants then completed the questionnaire individually, which took approximately 5–15 min. During the study period, approximately 2,000 patients attending the clinics met the provisional inclusion criteria. Owing to researcher availability constraints and clinical workload, only a subset of these eligible patients could be approached, and some patients declined to participate. Because the number of patients who were approached but declined was not systematically recorded, an exact response rate among those invited cannot be computed. A total of 408 patients completed the questionnaire. After quality screening, five questionnaires were excluded (two for logical inconsistencies and three for invariant responding/straight-lining), yielding a final analytical sample of 403 participants (valid questionnaire rate: 98.8%). A STROBE-style participant flow diagram illustrating this selection process is provided in Supplementary Material 1.

Inclusion criteria were: (1) a diagnosis of HIV/AIDS according to the Chinese Guidelines for the Diagnosis and Treatment of HIV/AIDS (2024 edition) (28), with clinically stable disease status (no active opportunistic infections in the past 3 months and receipt of stable antiretroviral therapy); (2) aged ≥ 18 years; and (3) able to communicate and complete the survey. Exclusion criteria were: (1) impaired consciousness or psychiatric disorders precluding cooperation; (2) severe physical illnesses (e.g., malignancies, organ failure); and (3) refusal to participate.

The study protocol was approved by the Ethics Committee of Zhongnan Hospital, Wuhan University (Approval No.: 2025206K), and the study was conducted in accordance with the Declaration of Helsinki. All participants provided written informed consent before participation.

### 2.2 Measures

#### 2.2.1 Demographic and clinical questionnaire

Participants self-reported demographic information (age, gender, education, occupation, monthly household income, marital status, sexual orientation) and clinical characteristics (time since HIV diagnosis, chronic comorbidities, HIV status disclosure). For analytic purposes, demographic and clinical variables were operationalized as follows: education was dichotomized as < college versus ≥ college; marital status as married versus unmarried (including divorced/widowed); sexual orientation as heterosexual, homosexual, bisexual, or other/uncertain; years since diagnosis as < 3 versus ≥ 3 years; chronic comorbidities as present versus absent; and HIV disclosure as disclosed versus not disclosed. Age and gender were retained in their original formats (continuous and male/female, respectively).

#### 2.2.2 Mindful attention awareness scale (MAAS)

The 15-item MAAS (29) assesses dispositional mindfulness on a 6-point Likert scale (1 = almost always to 6 = almost never). All items are reverse-scored; higher total scores (range 15–90) indicate greater mindfulness. The validated Chinese version (MAAS-C) (30) was used. Cronbach's α = 0.95 in this study.

#### 2.2.3 Brief resilience scale (BRS)

The 6-item BRS (31) assesses the ability to bounce back from stress on a 5-point scale (1 = strongly disagree to 5 = strongly agree). Three negatively worded items are reverse-scored; total scores range from 6 to 30. The validated Chinese version (BRS-C) (32) was used. Cronbach's α = 0.74.

#### 2.2.4 Generalized anxiety disorder-7 (GAD-7)

The 7-item GAD-7 (33) assesses anxiety symptoms over the past 2 weeks on a 4-point scale (0 = not at all to 3 = nearly every day). Total scores range from 0 to 21. The validated Chinese version (34) was used. Cronbach's α = 0.94.

#### 2.2.5 Patient health questionnaire-9 (PHQ-9)

The 9-item PHQ-9 (35) assesses depressive symptoms over the past 2 weeks on a 4-point scale (0 = not at all to 3 = nearly every day). Total scores range from 0 to 27. The validated Chinese version (36) was used. Cronbach's α = 0.92.

#### 2.2.6 World health organization quality of life-HIV, brief version (WHOQOL-HIV BREF)

The 31-item WHOQOL-HIV BREF (24) assesses quality of life across six domains: physical (4 items), psychological (5 items), independence (4 items), social relationships (4 items), environment (8 items), and spirituality/personal beliefs (4 items), plus two general items. Domain scores (range 4–20) and a total score (range 24–120) are calculated; higher scores indicate better quality of life. The validated Chinese version (37) was used. Cronbach's α = 0.82.

#### 2.2.7 Multidimensional scale of perceived social support (MSPSS)

The 12-item MSPSS (25) assesses perceived support from family, friends, and significant others (4 items each) on a 7-point scale (1 = strongly disagree to 7 = strongly agree). Total scores range from 12 to 84. The validated Chinese version (38) was used. Cronbach's α = 0.94.

### 2.3 Statistical analysis

#### 2.3.1 Latent profile analysis

Latent profile analysis (LPA) was performed in Mplus 8.3 using robust maximum likelihood estimation. Models with one to six profiles were estimated. GAD-7 and PHQ-9 were included as profile indicators alongside MAAS and BRS to capture distinct configurations of protective resources and distress symptoms, consistent with our focus on the resilience–distress paradox. Classification quality was evaluated using entropy and average posterior probabilities. Model selection was guided by lower AIC/BIC (39), significant LMR and BLRT tests, entropy >0.80, and a minimum profile size ≥ 5% (40).

#### 2.3.2 Profile comparisons

Following profile assignment, one-way ANOVAs examined profile differences in all psychological measures, quality of life, and social support. *Post hoc* comparisons used Bonferroni or Tamhane's T2 correction depending on Levene's test. Chi-square tests examined categorical sociodemographic variables; adjusted standardized residuals (ASRs) identified cells contributing to significant effects; following convention, cell-wise ASRs were evaluated against the ± 1.96 criterion without further multiplicity adjustment. Effect sizes (Cohen's d with 95% CI) were reported for all pairwise comparisons, particularly those involving the small Distressed profile. Effect sizes and their CIs were computed from unadjusted pairwise contrasts; *post-hoc p*-values were multiplicity-corrected.

#### 2.3.3 Supplementary moderation analysis

To complement the person-centered LPA, hierarchical multiple regressions tested the mindfulness × resilience interaction on anxiety and depression, modeled as separate dependent variables. All continuous predictors were mean-centered, and the interaction term was computed as the product of the centered scores. The primary models followed a two-step entry: Step 1 entered mindfulness and resilience as main effects; Step 2 added the mindfulness × resilience interaction term. Simple slopes were probed at the 16th, 50th, and 84th percentiles of mindfulness using the PROCESS macro (Model 1, 5,000 bootstrap samples) (41). In sensitivity analyses controlling for age, gender, chronic comorbidities, years since diagnosis, and education, the compensatory interaction pattern remained materially unchanged.

## 3 Results

### 3.1 Sample characteristics

Table 1 summarizes the demographic and clinical characteristics of the 403 participants. The mean age was 35.5 years (SD = 10.8), and the mean time since HIV diagnosis was 6.2 years (SD = 4.7). The majority were male (86.1%), unmarried (67.7%), and had disclosed their HIV status to others (69.7%). Approximately 70.2% reported no chronic comorbidities. Regarding specific conditions (with percentages calculated based on the total sample of 403 for overall prevalence), the most frequently reported chronic comorbidities among the 120 affected participants were cardiovascular diseases (8.2%, *n* = 33), endocrine/metabolic disorders (6.7%, *n* = 27), gastrointestinal diseases (5.7%, *n* = 23), and musculoskeletal conditions (5.5%, *n* = 22); categories were not mutually exclusive.

**Table 1:** Demographic and Clinical Characteristics of Participants (*N* = 403).

Variable	Category	*n*	%
Gender	Male	347	86.1
	Female	56	13.9
Education level	Primary school or below	41	10.2
	Junior high school	62	15.4
	High school/vocational	117	29.0
	College and above	183	45.4
Occupation	Professionals (e.g., teachers, doctors, lawyers, engineers)	104	25.8
	Self–employed/freelancers (e.g., writers, artists, photographers)	126	31.3
	Not in paid work (e.g., students, unemployed, retired, homemakers)	75	18.6
	Manual/service workers (e.g., workers, farmers, delivery personnel)	98	24.3
Monthly household income	No income	61	15.1
	≤ 3,000 yuan	48	11.9
	3,001–5,000 yuan	100	24.8
	5,001–10,000 yuan	137	34.0
	> 10,000 yuan	57	14.1
Marital status	Unmarried	273	67.7
	Married	94	23.3
	Divorced/widowed	36	8.9
Sexual orientation	Heterosexual	220	54.6
	Homosexual	104	25.8
	Bisexual	52	12.9
	Other/uncertain	27	6.7
Chronic comorbidities	No	283	70.2
	Yes	120	29.8
Disclosed HIV status to others	No	122	30.3
	Yes	281	69.7

Age: *M* = 35.5 ± 10.8 years; time since HIV diagnosis: *M* = 6.2 ± 4.7 years.

### 3.2 Latent profile analysis: model selection

Table 2 presents the model fit indices. The three-profile solution was selected as optimal. Although AIC, BIC, and aBIC continued to decrease through the six-profile model, the LMR test became non-significant for the four-profile (*p* = 0.096) and five-profile (*p* = 0.107) models, indicating no significant improvement over three profiles (39). The six-profile model yielded a significant LMR (*p* = 0.017) but its smallest profile contained only 1.95% of the sample (*n* = 8), below the 5% interpretability threshold (40). The three-profile model demonstrated high entropy (0.906) and excellent classification accuracy, with average posterior probabilities ranging from 0.93 to 0.97 across profiles. Its smallest profile (7.94%) exceeded the 5% criterion, ensuring substantive interpretability.

**Table 2:** Model fit indices for LPA of mindfulness, resilience, anxiety, and depression (*N* = 403).

Model	AIC	BIC	aBIC	Entropy	LMR (*p*)	BLRT (*p*)	Smallest profile (%)
1–Profile	10,689.437	10,721.429	10,696.044	—	—	—	100
2–Profile	10,178.360	10,230.346	10,189.096	0.820	0.0098	< 0.001	32.01
3–Profile	9,896.819	9,968.800	9,911.684	0.906	0.0091	< 0.001	7.94
4–Profile	9,804.340	9,896.316	9,823.335	0.912	0.0962	< 0.001	2.43
5–Profile	9,764.159	9,876.129	9,787.282	0.901	0.1069	< 0.001	4.01
6–Profile	9,727.434	9,859.399	9,754.687	0.913	0.0168	< 0.001	1.95

AIC, Akaike information criterion; BIC, Bayesian information criterion; aBIC, adjusted Bayesian information criterion; LMR, Lo–Mendell–Rubin likelihood ratio test; BLRT, bootstrap likelihood ratio test.

### 3.3 Profile characteristics

Figure 1 displays the standardized mean scores of the four indicator variables across profiles; Table 3 presents unstandardized means, standard deviations, and *post-hoc* comparisons. Profile 1—Well-Adapted (52.4%, *n* = 211)—was characterized by high mindfulness and resilience with minimal anxiety and depression, both well below clinical thresholds (GAD-7 < 5, PHQ-9 < 5). Profile 2—Moderate (39.7%, *n* = 160)—exhibited intermediate mindfulness and resilience with anxiety and depression in the mild-to-moderate clinical range. Profile 3—Distressed (7.9%, *n* = 32)—showed the lowest mindfulness and resilience, with anxiety in the severe range (GAD-7 ≥ 15) and depression approaching the severe threshold (PHQ-9 ≥ 20).

**Figure 1:**
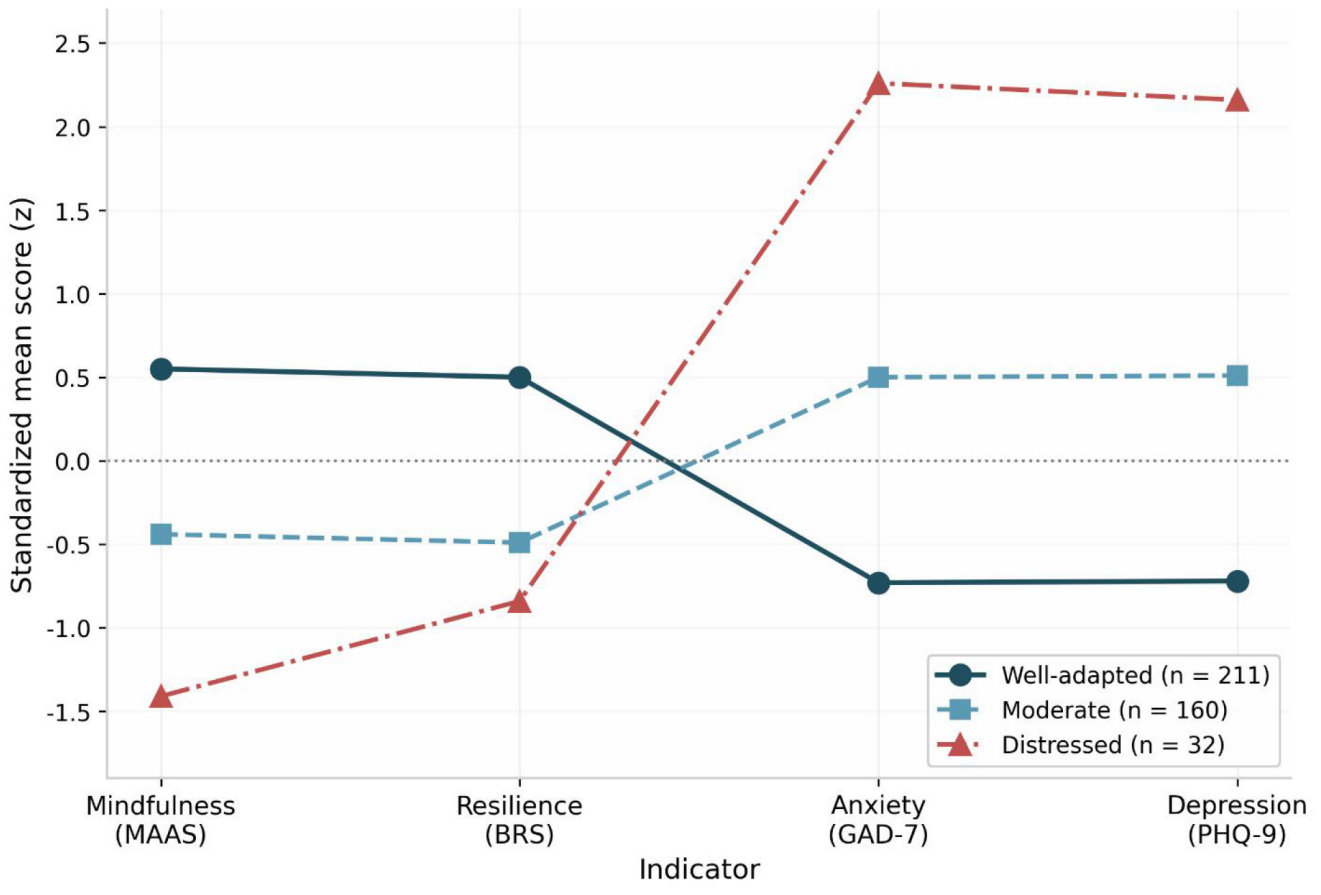
Standardized mean scores (*z*) of mindfulness, resilience, anxiety, and depression across the three latent profiles (Well-Adapted, Moderate, Distressed), computed relative to the total-sample mean and standard deviation of each indicator (*N* = 403).

**Table 3:** Comparison of psychological indicators across profiles (*M* ± *SD*).

Indicator	Well–Adapted (*n* = 211)	Moderate (*n* = 160)	Distressed (*n* = 32)	*F*	*p*	*Post–hoc*
Mindfulness (MAAS)	73.06 ± 12.00	57.84 ± 11.43	42.91 ± 15.22	128.76	< 0.001	*W > M > D*
Resilience (BRS)	21.93 ± 3.60	17.94 ± 3.30	16.50 ± 3.19	79.49	< 0.001	*W > M, D; M =* D
Anxiety (GAD−7)	1.22 ± 1.66	7.60 ± 2.92	16.72 ± 3.56	731.35	< 0.001	*D > M > W*
Depression (PHQ−9)	2.54 ± 2.59	9.87 ± 3.10	19.66 ± 4.81	581.68	< 0.001	*D > M > W*

*W*, Well–Adapted; *M*, Moderate; *D*, Distressed. All Levene's tests *p* < 0.05; Tamhane's T2 *post-hoc* tests were conducted.

All pairwise comparisons were significant (*p* < 0.001) with one exception: resilience did not significantly differ between the Moderate and Distressed profiles after Tamhane's T2 correction (adjusted *p* = 0.073), although the unadjusted comparison was nominally significant and the effect size was small-to-medium [*d* = 0.44, 95% CI (0.05, 0.83)]. However, given the small Distressed profile (*n* = 32), this comparison was likely underpowered; the non-trivial effect size alongside the non-significant corrected *p*-value indicates that the result should be read as inconclusive rather than as evidence of true equivalence.

### 3.4 Profile differences in quality of life

Table 4 presents the WHOQOL-HIV BREF domain scores and overall quality-of-life indices across the three profiles. All domains differed significantly (all *p* < 0.001). The Well-Adapted profile scored highest on every domain. The Moderate profile scored higher than the Distressed profile on all domains except social relationships and spirituality/personal beliefs, where differences were non-significant. The largest between-profile differences were in total quality of life (*F* = 121.12), psychological (*F* = 111.60), and spirituality/personal beliefs (*F* = 91.88) scores. Total quality of life followed a clear gradient: Well-Adapted (84.49 ± 10.90) > Moderate (70.05 ± 9.89) > Distressed (61.46 ± 12.07), all *p* < 0.001.

**Table 4:** Quality of life (WHOQOL–HIV BREF) by profile (*M* ± *SD*).

Domain	Well–Adapted (*n* = 211)	Moderate (*n* = 160)	Distressed (*n* = 32)	*F*	*p*	*Post-hoc*
Physical	14.26 ± 2.33	11.77 ± 2.44	10.38 ± 2.64	69.42	< 0.001	*W > M > D*
Psychological	14.54 ± 2.15	11.74 ± 1.99	10.33 ± 2.28	111.60	< 0.001	*W > M > D*
Independence	15.09 ± 2.29	13.03 ± 2.30	10.97 ± 2.79	63.80	< 0.001	*W > M > D*
Social relationships	13.12 ± 2.38	11.11 ± 2.56	10.31 ± 2.39	40.02	< 0.001	*W > M, D; M = D*
Environment	13.70 ± 2.17	11.93 ± 1.80	10.27 ± 2.34	58.99	< 0.001	*W > M > D*
Spirituality/personal beliefs	13.78 ± 2.73	10.48 ± 2.39	9.22 ± 3.20	91.88	< 0.001	*W > M, D; M = D*
Overall QoL total	84.49 ± 10.90	70.05 ± 9.89	61.46 ± 12.07	121.12	< 0.001	*W > M > D*
Health satisfaction (single item)	3.27 ± 0.87	2.66 ± 0.83	2.22 ± 0.75	36.51	< 0.001	*W > M > D*
Overall QoL perception (single item)	3.70 ± 0.81	3.06 ± 0.58	2.63 ± 0.79	53.66	< 0.001	*W > M > D*

All post–hoc comparisons Bonferroni–corrected at α = 0.05 (Levene's *p* ≥0.05 for all quality–of–life domains). Overall QoL total = sum of domain scores.

### 3.5 Profile differences in social support

Table 5 presents MSPSS scores across profiles. Total support differed significantly across profiles, F (2, 400) = 10.33, *p* < 0.001, as did all three subscales (Family: *F* = 7.57, *p* =0.001; Friends: *F* = 11.54, *p* < 0.001; Significant other: *F* = 4.38, *p* = 0.013). Bonferroni-corrected *post-hoc* comparisons showed that the Well-Adapted profile scored significantly higher than the Moderate profile on total support [mean difference = 7.79, corrected *p* < 0.001, *d* = 0.47, 95% CI (0.26, 0.68)] and on every subscale (corrected ps ≤ 0.011). In contrast, no comparison involving the Distressed profile survived correction: Well-Adapted vs. Distressed [corrected *p* = 0.127, *d* = 0.38, 95% CI (−0.01, 0.75)] and Moderate vs. Distressed [corrected *p* = 1.000, *d* = −0.08, 95% CI (−0.46, 0.30)]. Notably, the Well-Adapted–Distressed difference in total support was nominally significant before correction (uncorrected *p* = 0.042), and the Friends subscale comparison approached significance even after correction (*p* = 0.051); given the small Distressed profile (*n* = 32), these patterns likely reflect insufficient statistical power rather than true equivalence.

**Table 5:** Social support (MSPSS) by profile (*M* ± *SD*).

Source	Well–Adapted (*n* = 211)	Moderate (*n* = 160)	Distressed (*n* = 32)	*F*	*p*	*Post–hoc*
Family	19.96 ± 6.58	17.24 ± 6.76	17.78 ± 8.58	7.57	0.001	*W > M*
Friends	19.74 ± 6.34	16.66 ± 6.31	16.84 ± 6.84	11.54	< 0.001	*W > M*
Significant other	20.87 ± 6.26	18.89 ± 6.47	19.47 ± 7.84	4.38	0.013	*W > M*
Total	60.58 ± 16.46	52.79 ± 16.60	54.09 ± 19.50	10.33	< 0.001	*W > M*

All *post-hoc* comparisons Bonferroni–corrected at α =0.05 (Levene's *p* ≥0.05 for all MSPSS scales).

Sensitivity analyses excluding the Distressed profile confirmed the robustness of the Well-Adapted–Moderate gradient: all social support differences remained significant (total support: *p* < 0.001; subscales: all ps ≤ 0.003), and the quality-of-life pattern was unchanged.

### 3.6 Profile differences in sociodemographic and clinical variables

Table 6 presents the distribution of sociodemographic and clinical variables across profiles. Sexual orientation distribution differed significantly across profiles (χ^2^ = 11.60, df = 4, *p* = 0.021). Adjusted standardized residual (ASR) analysis indicated that, within the Distressed profile, the observed frequency of heterosexual individuals was significantly lower than expected (ASR = −3.13, *p* < 0.01), whereas homosexual individuals were significantly overrepresented (ASR = 2.00, *p* < 0.05); the other/uncertain category showed a non-significant trend toward overrepresentation (ASR = 1.73). No cells in the Well-Adapted or Moderate profiles reached statistical significance (all |ASR| < 1.96). Years since diagnosis differed significantly across profiles (χ^2^ = 11.90, df = 2, *p* =0.003). Adjusted standardized residual analysis indicated that the Well-Adapted profile contained significantly fewer recently diagnosed individuals than expected (ASR = −3.26, *p* < 0.01), whereas both the Moderate (ASR = 2.17, *p* < 0.05) and the Distressed (ASR = 2.10, *p* < 0.05) profiles contained significantly more recently diagnosed individuals than expected. No other sociodemographic or clinical variables—including age, gender, education, occupation, income, marital status, chronic comorbidities, and HIV status disclosure—differed significantly across profiles (all *p* >0.05).

**Table 6:** Sociodemographic and clinical variables by profile.

Variable	Well–Adapted (*n* = 211)	Moderate (*n* = 160)	Distressed (*n* = 32)	Test statistic	*p*
Age (years), M ± SD	36.4 ± 11.3	34.4 ± 10.1	35.0 ± 10.3	*F* = 1.61	0.201
Gender (male), *n* (%)	186 (88.2)	137 (85.6)	24 (75.0)	χ^2^ = 4.07	0.131
Education (≥ college), *n* (%)	100 (47.4)	69 (43.1)	14 (43.8)	χ^2^ = 0.74	0.691
Married, *n* (%)	54 (25.6)	30 (18.8)	10 (31.3)	χ^2^ = 3.60	0.160
Sexual orientation, *n* (%)				χ^2^ = 11.60	0.021
Heterosexual	115 (54.5)	96 (60.0)	9 (28.1)^a^		
Homosexual	52 (24.6)	39 (24.4)	13 (40.6)^a^		
Other/uncertain	44 (20.9)	25 (15.6)	10 (31.3)		
Years since diagnosis, *n* (%)				χ^2^ = 11.90	0.003
< 3 years	44 (20.9)^a^	54 (33.8)^a^	14 (43.8)^a^		
≥3 years	167 (79.1)	106 (66.2)	18 (56.2)		

Bisexual participants (*n* = 52) were collapsed into the ‘Other/uncertain' category due to small cell sizes to meet chi–square test assumptions. No significant differences were observed across profiles in occupation (χ^2^ = 9.21, *p* = 0.162), monthly income (χ^2^ = 8.97, *p* = 0.345), chronic comorbidities (χ^2^ = 1.66, *p* = 0.436), or HIV disclosure (χ^2^ = 0.23, *p* = 0.890). ^a^ |ASR| > 1.96, *p* < 0.05. For years since diagnosis (a 3 × 2 table), the ≥ 3 years cells carry ASRs of equal magnitude and opposite sign and are therefore not separately flagged.

### 3.7 Supplementary moderation analysis: mindfulness × resilience interaction

Hierarchical multiple regressions tested whether mindfulness moderated the resilience–distress relationship. Predictors were mean-centered, and the interaction term was the product of the centered scores. For anxiety (GAD-7), the full model including mindfulness, resilience, and their interaction was significant, *R*^2^ =0.414, *F* (3, 399) = 94.08, *p* < 0.001, explaining 41.4% of the variance; the mindfulness × resilience interaction significantly predicted anxiety, Δ*R*^2^ = 0.013, B = 0.010, *p* = 0.003, 95% CI [0.004, 0.017]. For depression (PHQ-9), the full model was likewise significant, *R*^2^ = 0.445, *F* (3, 399) = 106.82, *p* < 0.001, explaining 44.5% of the variance; the interaction term significantly predicted depression, Δ*R*^2^ = 0.010, B = 0.010, *p* = 0.006, 95% CI [0.003, 0.018].

Simple slope analyses (Figure 2) were conducted at the 16th, 50th, and 84th percentiles of mindfulness (centered values = −14.63, −0.63, and 17.37, respectively). Resilience significantly predicted lower distress at all levels of mindfulness (all *p* < 0.05). The protective effect was stronger at low mindfulness (anxiety: effect = −0.503, *p* < 0.001; depression: effect = −0.505, *p* < 0.001) than at high mindfulness (anxiety: effect = −0.177, *p* = 0.022; depression: effect = −0.172, *p* = 0.047), with intermediate effects at median mindfulness (anxiety: effect = −0.361, *p* < 0.001; depression: effect = −0.359, *p* < 0.001). At low mindfulness, predicted anxiety decreased from 8.45 at low resilience to 4.92 at high resilience, whereas at high mindfulness the corresponding change was only from 2.58 to 1.34; likewise, predicted depression decreased from 10.93 to 7.39 at low mindfulness but only from 3.60 to 2.40 at high mindfulness. This pattern indicates a compensatory interaction in which resilience provides greater incremental benefit when mindfulness is depleted.

**Figure 2:**
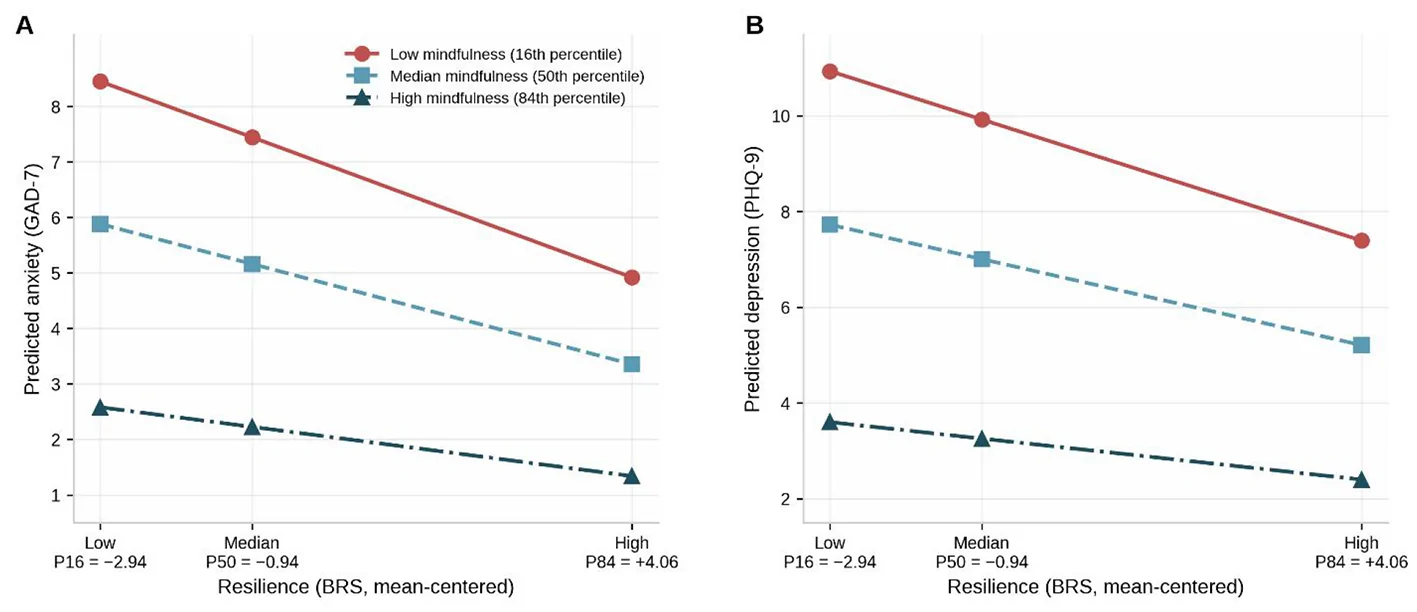
Simple slopes of resilience on anxiety **(A)** and depression **(B)** at low (16th percentile), median (50th percentile), and high (84th percentile) levels of mindfulness. The *x*-axis represents mean-centered resilience (BRS), with tick marks at its 16th (P16 = −2.94), 50th (P50 = −0.94), and 84th (P84 = +4.06) percentiles; these values are asymmetric around zero because percentile-based probing was used. All simple slopes were significant (*p* < 0.05), and the steeper negative slopes at low mindfulness support a compensatory interaction pattern. *N* = 403.

## 4 Discussion

### 4.1 Discrete psychological adaptation profiles and the resilience–distress paradox

We identified three discrete psychological adaptation profiles among PLWH: Well-Adapted (52.4%), Moderate (39.7%), and Distressed (7.9%). Consistent with the person-oriented premise that mental health reflects organized configurations rather than a linear continuum (7), these profiles confirm that the relationship between protective factors and psychological distress varies across profiles, further underscoring the value of a person-centered approach (8).

The Moderate profile exhibited subclinical anxiety and depression, yet already demonstrated a substantially lower quality of life than the Well-Adapted profile. This mismatch between subclinical symptoms and notable functional impairment carries important clinical implications: under dichotomous screening, this large profile (39.7%) would risk being overlooked despite its evident functional decline. Prior LPA studies in PLWH have consistently identified a “moderate” profile qualitatively distinct from both low- and high-severity profiles (19, 20). Collectively, these findings establish the Moderate profile as a high-yield target for secondary prevention and early intervention: timely intervention before symptoms escalate may prove more cost-effective and may prevent progression toward the Distressed profile.

Most strikingly, the Moderate and Distressed profiles did not differ significantly in resilience after correction for multiple comparisons—both fell within the “low resilience” range—yet their distress levels diverged sharply, with the Distressed profile scoring well within the clinical range on both anxiety and depression. The distinguishing factor was instead mindfulness: mindfulness declined monotonically across profiles, whereas resilience failed to differentiate the latter two. This pattern suggests a potential threshold effect of mindfulness: when mindfulness capacity is low or underdeveloped, resilience alone may no longer suffice to buffer distress.

Notably, the Distressed profile contained a significantly higher proportion of individuals diagnosed within the past 3 years (43.8%) compared with the Well-Adapted profile (20.9%). This pattern may reflect the acute psychological shock of recent diagnosis, before adaptive coping mechanisms have consolidated, or conversely, a survivor effect whereby longer-term PLWH have developed greater psychological accommodation to chronic illness. Longitudinal research is needed to disentangle whether this association represents a time-limited adjustment reaction or a selection bias toward persistent distress. Taken together, these profile characteristics suggest that the Distressed group is marked not only by depleted psychological resources but also by limited time for adaptation. We examine the resource-interaction hypothesis directly in the next section.

### 4.2 Mindfulness and resilience: a compensatory interaction pattern

The resilience × mindfulness interaction was significant for both anxiety and depression. Simple-slope tests revealed that resilience predicted lower distress across all levels of mindfulness, yet its protective effect was substantially stronger at low than at high mindfulness. These findings argue against a threshold model and instead support a compensatory pattern: resilience confers its greatest benefit precisely when mindfulness is most depleted (Figure 2).

This compensatory pattern is consistent with resource substitution theories: when one protective factor is deficient, another can step in to compensate. Individuals low in mindfulness may lack the decentering and nonreactivity that normally attenuate distress (13), and may therefore rely more heavily on resilience-based strategies such as active coping and cognitive reappraisal (10). When mindfulness is high, individuals already regulate emotions through present-moment awareness and acceptance (42), leaving limited additional room for benefit from resilience. The LPA results corroborate this model: the Moderate profile, despite low resilience, remained at subclinical distress levels through moderate mindfulness; the Distressed profile, in which both resources were depleted, lacked any compensatory buffer; and the Well-Adapted profile, with high levels of both, reported minimal distress. Thus, mindfulness and resilience can partially compensate for each other, whereas the concurrent absence of both resources creates the greatest vulnerability.

This interaction reflects diminishing returns rather than interference: because mindfulness itself was a strong direct predictor of distress, the incremental contribution of resilience was necessarily smaller among high-mindfulness individuals—not because mindfulness impairs resilience in any way, but because the marginal benefit of an additional resource diminishes when the primary resource is already abundant. This interpretation converges with prior evidence that identical levels of resilience can produce divergent outcomes depending on co-occurring factors (23), that mindfulness and resilience jointly predict anxiety in PLWH (11), and that mindfulness differentiates psychological profiles (43) in part by enhancing distress tolerance and reducing cognitive fusion (44). Taken together, these findings point to a stratified intervention logic in which the optimal treatment target depends on an individual's mindfulness level—a framework we develop in the following paragraph.

This conditional relationship may be especially pronounced in PLWH, who face unique stressors including HIV-related stigma, treatment burden, and existential distress. Mindfulness may help individuals decouple their self-concept from their illness identity, thereby preventing internalized stigma from eroding resilience. These findings suggest a preliminary framework for stratified intervention: for the Moderate profile, mindfulness-based stress reduction may enhance the capacity to leverage existing resilience; for the Distressed profile, an integrated intervention combining mindfulness training with identity-affirming support may be warranted. In particular, whether resilience training can be delivered directly to individuals low in mindfulness—without first building mindfulness capacity—merits rigorous evaluation in longitudinal or experimental designs. The following section examines sexual orientation differences and provides additional support for this conditional pattern from a social-identity perspective.

We acknowledge that framing resilience as an individual capacity risks placing the burden of adaptation on PLWH themselves—a critique well-established in resilience scholarship, particularly for marginalized populations (45, 46). This concern is especially salient for our sample, which includes sexual-minority individuals facing intersecting stigma. Resilience should not be invoked to justify inaction on the systemic stigma, discrimination, and structural inequities that produce adversity in the first place. Future interventions must therefore address both individual-level resources and the structural barriers that erode them.

### 4.3 Sexual orientation and identity uncertainty: findings requiring cautious interpretation

Sexual orientation distribution differed significantly across the three profiles. Cell-wise analysis identified two statistically reliable patterns within the Distressed profile: a significant underrepresentation of heterosexual individuals and a significant overrepresentation of homosexual individuals, the latter consistent with our *a priori* prediction derived from minority stress theory. The other/uncertain category also showed a trend toward overrepresentation that did not reach statistical significance. Thus, the overall association was driven jointly by the underrepresentation of heterosexual individuals and the overrepresentation of homosexual individuals in the Distressed profile. Given the small cell frequencies involved, these findings nonetheless warrant replication, and the interpretation of the identity-uncertain trend remains hypothesis-generating.

Although the overrepresentation of identity-uncertain individuals in the Distressed profile did not reach statistical significance, its direction is consistent with the following theoretical account. Individuals who are exploring or uncertain about their sexual orientation lack the identity safety afforded by heteronormativity. This state of “identity suspension” may continuously deplete cognitive and emotional resources. Drawing on identity development theory (47) and the minority stress model (26), chronic identity negotiation may tax working memory and inhibitory control, progressively draining the cognitive resources needed for emotion regulation. Consistent with this account, minority stress has been linked to poorer cognitive performance, particularly fluid intelligence, in sexual-minority older adults (48). One speculative possibility is that, when internal resource depletion is compounded by external minority stressors (e.g., internalized stigma, anticipated discrimination), insufficient mindfulness leaves resilience unable to buffer the accumulated distress. This interpretation is broadly consistent with evidence that identity-related cognitive load consumes attentional resources (49), and that unresolved identity issues—such as identity uncertainty—are associated with elevated psychological distress in sexual-minority samples (50).

It is important to emphasize that this study did not directly measure internalized homophobia, identity distress, outness, minority stress experiences, or LGBTQ+ community belongingness, and that cell frequencies within the Distressed profile were small (9–13 participants per category); in particular, the overrepresentation of identity-uncertain individuals did not reach statistical significance, and the effect for homosexual participants reached the conventional threshold (ASR = 2.00, *p* < 0.05) but was marginal and should be interpreted cautiously given the small cell size. Consequently, although the underrepresentation of heterosexual individuals and the overrepresentation of homosexual individuals were statistically reliable, all interpretations regarding sexual orientation and psychological distress remain non-causal and require replication in larger samples.

The observed association could also reflect reverse causality (e.g., distress exacerbating identity confusion) or unmeasured confounders (e.g., social rejection, internalized stigma), both of which are well-established minority stressors linked to adverse mental health outcomes (27). Given the small absolute frequencies in the Distressed profile, these estimates—including the marginally significant overrepresentation of homosexual participants—are sensitive to minor distributional shifts and should be replicated before being considered robust. Future longitudinal studies are needed to track identity commitment trajectories, and qualitative methods could help disentangle the distinct effects of internalized stigma, social rejection, and identity uncertainty on psychological adaptation. Additionally, future research with larger sexual-minority-specific samples should examine whether the three-profile structure replicates in this subpopulation and whether the compensatory interaction pattern differs by sexual orientation.

### 4.4 Social support: perceived availability vs. functional utilization

Perceived social support (MSPSS total) exhibited a partial hierarchical pattern: it differentiated the Well-Adapted from the Moderate profile, but neither differed significantly from the Distressed profile.

This pattern, however, must be interpreted with caution. The small subsample of the Distressed profile limited statistical power to detect small-to-medium effects: the Well-Adapted–Distressed difference, though non-significant, was of modest magnitude (*d* = 0.38), and is best interpreted as inconclusive rather than as evidence of no difference—a substantial risk of Type II error. Notably, the Moderate and Distressed profiles showed virtually no difference in perceived social support, suggesting that perceived availability alone does not distinguish moderate from severe distress.

Conceptually, the MSPSS captures the perceived availability of support rather than its functional mobilization during acute psychological crises (25). Meta-analytic evidence shows that perceived and received support are only moderately correlated (51) and differentially predict mental health outcomes. In particular, perceived support consistently outperforms received support in predicting psychological adjustment—a finding confirmed across a synthesis of 60 meta-analyses (52). Individuals with severe distress often exhibit a dissociation between intact support networks and actual support mobilization, with rumination as a key mechanism underlying this impairment, because it hinders effective problem-solving and help-seeking (53). Consequently, the critical distinction between moderate and severe distress may lie not in structural availability alone, but in support mobilization capacity—the ability to actively access and benefit from available resources during crises. Future research should move beyond cross-sectional self-reports and adopt multimodal assessment paradigms, such as ecological momentary assessment, to capture the dynamic interplay between perceived availability and actual support utilization in real time (54).

### 4.5 Limitations

This study has several limitations. First, the cross-sectional design precludes causal inference and the establishment of temporal sequences; interpretations of mindfulness as a context-dependent moderator therefore remain hypothesis-generating and require longitudinal or experimental validation. Second, the substantial gender imbalance (86.1% male) constrains generalizability: the identified profile structure and its correlates may not extend to women or gender-diverse PLWH, and future studies should test measurement invariance across genders. Third, the exclusive reliance on self-report measures at a single time point leaves the observed associations vulnerable to common method bias; multi-informant and longitudinal designs are needed. Fourth, the small Distressed subsample afforded limited power for *post hoc* comparisons involving this profile; non-significant findings pertaining to the Distressed profile should therefore be read as inconclusive rather than as evidence of no group difference. Fifth, key HIV clinical indicators (e.g., CD4 count, viral load) and psychosocial treatment history were not assessed and may constitute unmeasured confounders. Sixth, selection bias may further limit generalizability. Although approximately 2,000 eligible patients were identified during the study period, only 408 were recruited owing to researcher availability and clinical workload. We did not systematically record the number of eligible patients missed or declining participation. This non-random selection from the eligible pool may have biased the sample—for instance, patients with greater psychological distress may have been either more or less likely to be available or willing to participate. Accordingly, the reported profile prevalences should be interpreted with caution.

### 4.6 Conclusion

This study demonstrates that psychological adaptation among PLWH is highly heterogeneous. Using latent profile analysis, we identified three distinct psychological adaptation profiles—Well-Adapted, Moderate, and Distressed—each characterized by a unique configuration of mindfulness, resilience, anxiety, and depression. A compensatory rather than synergistic pattern emerged: interaction analyses revealed that resilience compensates for low mindfulness, conferring its greatest protection precisely where mindfulness is most depleted. Contextualized within the profile results, this compensatory pattern offers a direct account of the resilience–distress paradox, illustrating how comparable resilience can yield markedly different distress outcomes depending on mindfulness availability. These findings challenge purely additive or threshold models of protective factors and support resource substitution as a more parsimonious account. Tailoring interventions to an individual's resource profile may improve treatment efficacy. Given the cross-sectional design, causal inferences remain tentative, and the compensatory model awaits replication in independent samples.

## Data Availability

The raw data supporting the conclusions of this article will be made available by the authors, without undue reservation.
