# Correction: The resilience–distress paradox in HIV: latent profiles reveal that resilience compensates for low mindfulness

**DOI:** 10.3389/fpubh.2026.2028306

**Published:** 2026-10-30

**Authors:** Fang Chen, Xixi Yan, Xiaomin Ding, Juan Li, Shihua Pan, Ling Feng, Xiaoxia Zhang, Yueling Wu, Huimin Sun, Hongyun Xu

**Affiliations:** 1 Department of Neurology, Zhongnan Hospital of Wuhan University, Wuhan, China; 2 Department of Allergy, Zhongnan Hospital of Wuhan University, Wuhan, China; 3 Department of Geriatrics, Zhongnan Hospital of Wuhan University, Wuhan, China; 4 Medical Social Work Department, Zhongnan Hospital of Wuhan University, Wuhan, China; 5 Department of General Medicine, Zhongnan Hospital of Wuhan University, Wuhan, China; 6 Department of Infectious Diseases, Zhongnan Hospital of Wuhan University, Wuhan, China; 7 Department of Scientific Research, Zhongnan Hospital of Wuhan University, Wuhan, China; 8 Department of Neuropsychology, Zhongnan Hospital of Wuhan University, Wuhan, China; 9 Party Committee Office, Zhongnan Hospital of Wuhan University, Wuhan, China

**Keywords:** compensatory interaction, HIV/AIDS, latent profile analysis, mindfulness, psychological distress, quality of life, resilience

There was a mistake in the images of Figures 1, 2 as published. The image for Figure 1 was the one for Figure 2 and vice versa. The corrected Figures 1, 2 and their captions appear below.

**Figure 1:**
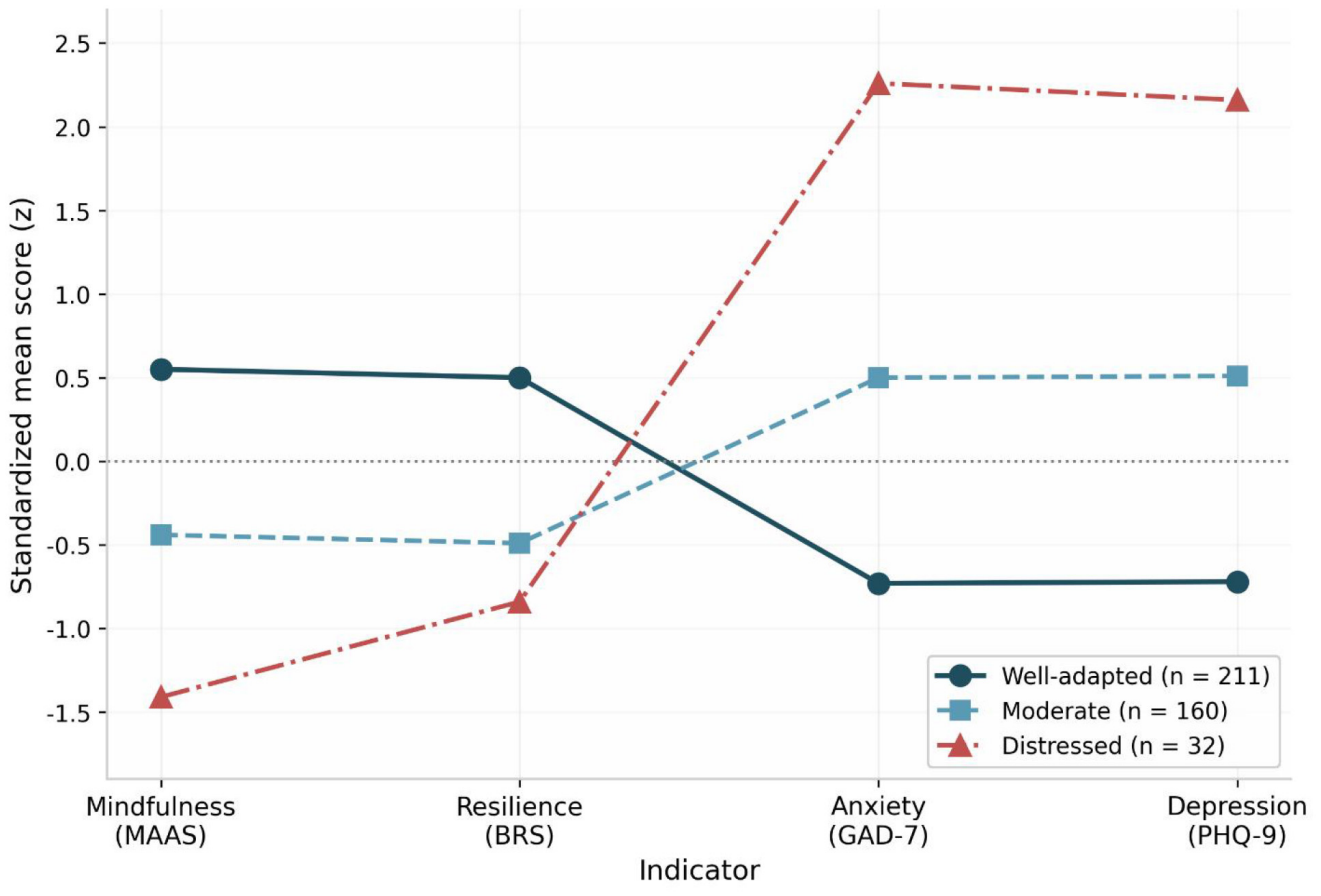
Standardized mean scores (*z*) of mindfulness, resilience, anxiety, and depression across the three latent profiles (Well-Adapted, Moderate, Distressed), computed relative to the total-sample mean and standard deviation of each indicator (*N* = 403).

**Figure 2:**
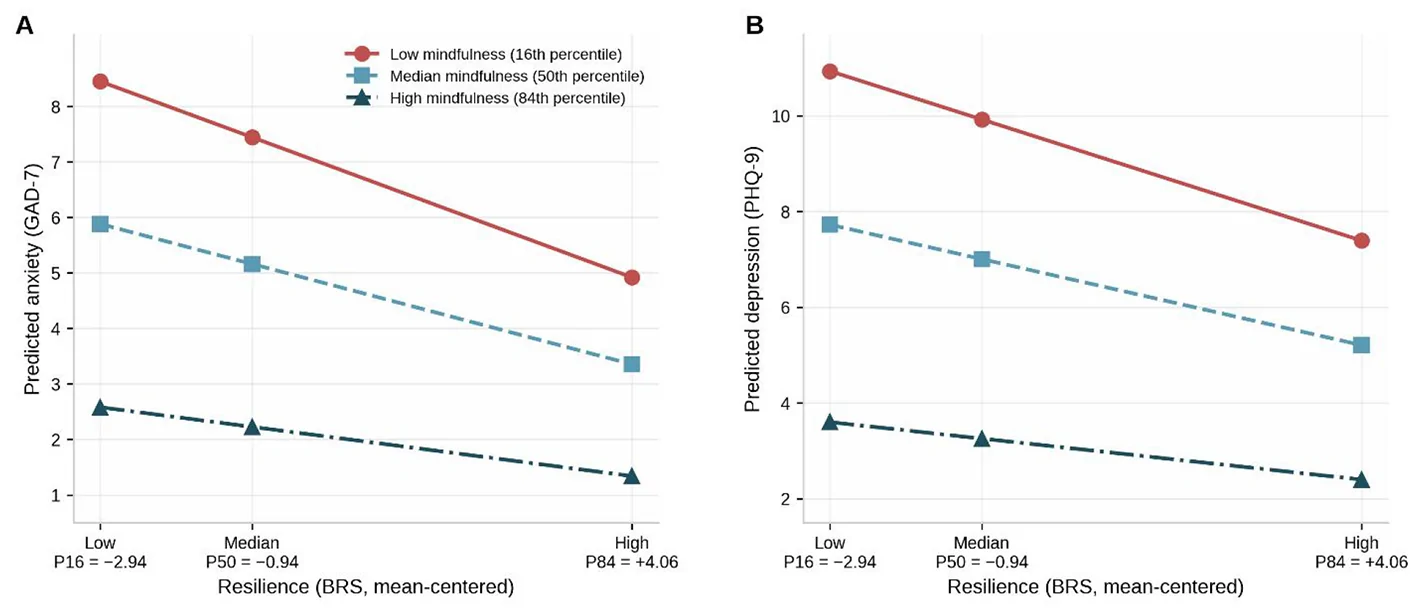
Simple slopes of resilience on anxiety **(A)** and depression **(B)** at low (16th percentile), median (50th percentile), and high (84th percentile) levels of mindfulness. The *x*-axis represents mean-centered resilience (BRS), with tick marks at its 16th (P16 = −2.94), 50th (P50 = −0.94), and 84th (P84 = +4.06) percentiles; these values are asymmetric around zero because percentile-based probing was used. All simple slopes were significant (*p* < 0.05), and the steeper negative slopes at low mindfulness support a compensatory interaction pattern. *N* = 403.

In addition, the following typographical errors are corrected. In reference 34, a period was missing between the article title and the journal name; the reference should read “34. Qu S, Sheng L. Diagnostic test of screening generalized anxiety disorders in general hospital psychological department with GAD-7. *Chin Ment Health J*. (2015) 29:939–44. doi: 10.3969/j.issn.1000-6729.2015.12.010.” In the title of **Table 5**, “by Profile” should read “by profile.” **Table 5**. Social support (MSPSS) by profile (*M* ± SD). In reference 37, the page range should read “16:1–2, 52” instead of “16:1–2.”; the reference should read 37. Liu W, He L, Wang J, Liu B, van Ommeren M, Fang J, et al. Introduction and instructions for the Chinese version of the WHOQOL-HIV BREF. *China J Tradit Chin Med Inf*. (2009) 16:1–2, 52. doi: 10.3969/j.issn.1005-5304.2009.10.001.

The original version of this article has been updated.

## Generative AI statement

Any alternative text (alt text) provided alongside figures in this article has been generated by Frontiers with the support of artificial intelligence and reasonable efforts have been made to ensure accuracy, including review by the authors wherever possible. If you identify any issues, please contact us.

