# Supplementary material for: The resilience–distress paradox in HIV: latent profiles reveal that resilience compensates for low mindfulness
Source: Front Public Health. 2026 Oct 12;14:1950568. doi: 10.3389/fpubh.2026.1950568 (PMC13696326; doi:10.3389/fpubh.2026.1950568)
Supplement: Supplementary file 1 [file Data_Sheet_1.pdf]

Supplementary Material 1

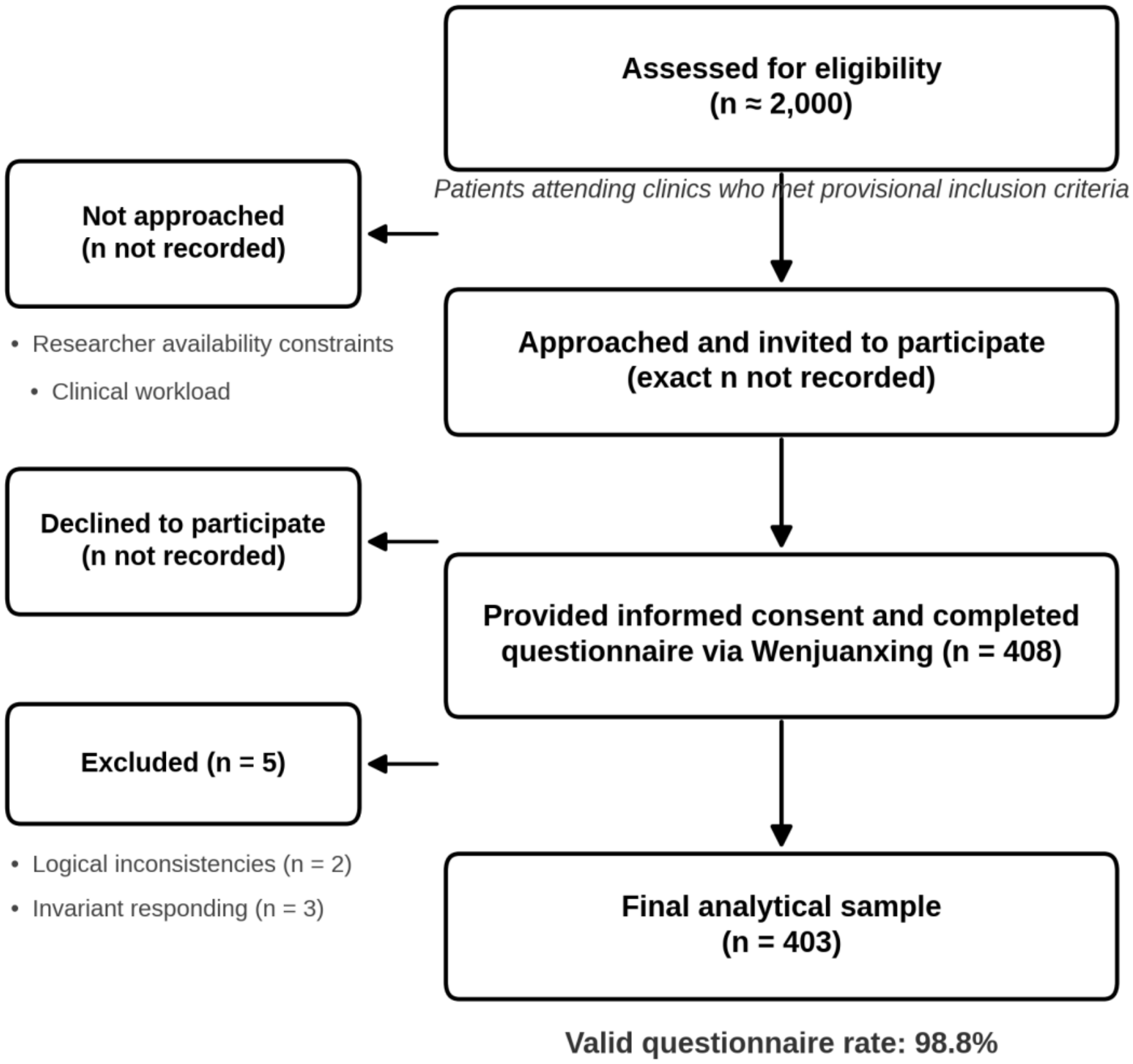

Note. The exact numbers of eligible patients who were not approached (owing to researcher availability constraints and clinical workload) and of those who declined participation were not systematically recorded; therefore, an exact response rate among invitees cannot be computed.

Study period: December 2025 – March 2026

Sites: Zhongnan Hospital & Wuhan Jinyintan Hospital, Wuhan, China
